# Predicting implantation by using dual AI system incorporating three‐dimensional blastocyst image and conventional embryo evaluation parameters—A pilot study

**DOI:** 10.1002/rmb2.12612

**Published:** 2024-09-30

**Authors:** Yasunari Miyagi, Toshihiro Habara, Rei Hirata, Nobuyoshi Hayashi

**Affiliations:** ^1^ Medical Data Labo Okayama City Okayama Prefecture Japan; ^2^ Okayama Couple's Clinic Okayama City Okayama Prefecture Japan

**Keywords:** artificial intelligence, assisted reproductive technology, blastocyst, implantation, time lapse

## Abstract

**Purpose:**

To investigate the usefulness of an original dual artificial intelligence (AI) system, in which the first AI system eliminates the background of sliced tomographic blastocyst images, then the second AI system predicts implantation success using three‐dimensional (3D) reconstructed images of the sequential images and conventional embryo evaluation parameters (CEE) such as maternal age.

**Methods:**

Patients (from June 2022 to July 2023) could opt out and there was additional information on the Web site of the clinic. Implantation and non‐implantation cases numbered 458 and 519, respectively. A total of 10 747 tomographic images of the blastocyst in a time‐lapse incubator system with the CEE were obtained.

**Results:**

The statistic values by the dual AI system were 0.774 ± 0.033 (mean ± standard error) for area under the characteristic curve, 0.727 for sensitivity, 0.719 for specificity, 0.727 for predictive value of positive test, 0.719 predictive value of negative test, and 0.723 for accuracy, respectively.

**Conclusions:**

The usefulness of the dual AI system in predicting implantation of blastocyst in handling 3D data with conventional embryo evaluation information was demonstrated. This system may be a feasible option in clinical practice.

## Graphical abstract

We made an original dual artificial intelligence (AI) system, in which the first AI system eliminates the background of sliced tomographic blastocyst images with using a time‐lapse device, then the second AI system predicts implantation success using three‐dimensional 3D reconstructed images of the sequential images and conventional embryo evaluation parameters such as maternal age. The statistic values by the dual AI system were 0.774 ± 0.033 (mean ± standard error) for area under the characteristic curve. The usefulness of the dual AI system in predicting implantation was demonstrated.

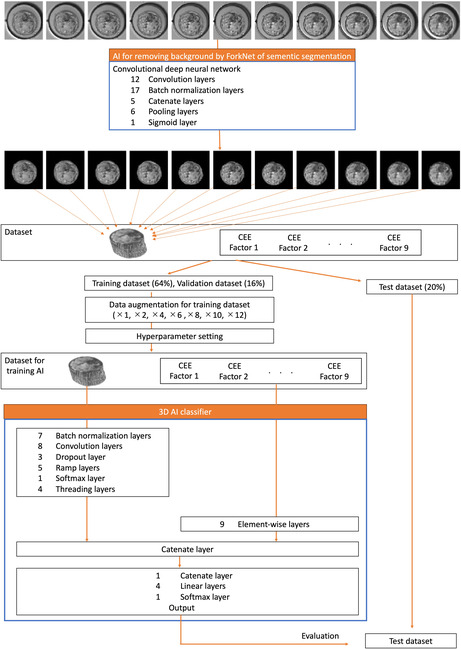

## 1 INTRODUCTION

While grading systems have been used for some time such as the Gardner grading system,^1^ artificial intelligence (AI)‐based methods have recently been reported for evaluating blastocysts in assisted reproductive technology. A method using time‐lapse imaging for evaluating implantation from multiple images has also been reported. Time‐lapse imaging, recently used in many facilities, is a system for observing information over time in blastocysts, and there are reports of the results of AI analysis of a blastocyst's time course information. The area under the characteristic curve (AUC) for implantation prediction by deep learning has been reported as 0.736^2^ and 0.71^3^ (Table 1). The AUC range for predicting implantation by deep learning was found to be 0.662–0.759 classified by age.^4^ The AUC for predicting implantation was 0.74 for a proposed random forest model.^5^ A predictive model of implantation constructed based on principal component analysis and AI showed an AUC of 0.75.^6^ The performance of an algorithm with AI for morphometric parameters of blastocysts using time‐lapse video had an AUC of 0.70 for improving blastocyst selection.^7^ There is a multicenter, double‐blind, randomized controlled trial report that the use of time‐lapse imaging systems for embryo culture and selection did not significantly increase the odds of live birth compared with standard care without time‐lapse imaging.^8^ Thus, the usefulness of time observation for time‐lapse systems has not been established, it would be worth exploring other approaches.

**TABLE 1:** Comparison of published reports of the AUC for predicting implantation.

The AUC^a^ values	Author (published year)
0.75^6^	Milewski (2017)
0.74^5^	Blank (2019)
0.662–0.759^4^	Ueno (2021)
0.71^3^	Enatsu (2022)
0.70^7^	Fruchter‐Goldmeier (2023)
0.736^2^	Ueno (2024)
0.774 ± 0.033	Miyagi (2024, this study)

^a^ Area under the characteristic curve.

As AI can learn various types of multimodality information,^9^, ^10^, ^11^, ^12^ conventional embryo evaluation (CEE) parameters such as maternal age, in addition to time‐lapse information, are also being applied in reproductive medicine. We reported (2020) an AUC of 0.74 for predicting live births by AI generated from single images alone and AI generated from single images plus CEE parameters.^12^ Salih et al.^13^ reported that the best‐performing prediction models for clinical outcomes combined clinical information and images including time‐lapse video. In a combined time‐lapse imaging and CEE report, the average AUC of a time‐lapse video and clinical data model was 0.727.^14^

Depth imaging is possible with some models because embryo culture in time‐lapse instruments is performed in dedicated wells. Information from time‐lapse instruments has usually been interpreted and used in terms of time, but we applied these instruments for reconstructing 3D image data by depth imaging and used this as spatial information for AI training together with CEE.

In the present study, we created the first original AI system to eliminate the background of multiple cross‐sectional images of blastocysts sliced with a fixed pitch width just before freezing. We did this using a time‐lapse device and by using a model based on a semantic segmentation.^15^ We then created a second original AI system to analyze the spatial information of the blastocysts in terms of implantation prediction, using 3D data of multiple cross‐sections constructed on a computer and with nine CEE parameters. We were able to show good implantation prediction performance with this dual AI system.

## 2 MATERIALS AND METHODS

### 2.1 Patients and data preparation

This study collected images of blastocysts with morphological features and clinical information. These were obtained from consecutive patients at the Okayama Couples' Clinic (Okayama, Japan) between June 18, 2022, and July 31, 2023, with completely deidentified data. Only elective single embryo transfer was performed. All blastocysts were tracked to confirm whether the result was an implantation or non‐implantation. This retrospective study was approved by the Institutional Review Board (IRB) of Okayama Couples' Clinic (IRB number 2022‐09). This non‐interventional study gave patients the chance to opt out and to obtain additional information on the clinic's Web site.

### 2.2 Conventional embryo evaluation

All blastocysts with clinical information and morphological features, including maternal age, body mass index (BMI), number of previous embryo transfer procedures, in vitro fertilization time, anti‐Müllerian hormone concentration, follicle‐stimulating hormone value, blastocyst grade on day 3, embryo cryopreservation day, trophectoderm grade, inner cell mass grade, number of blastomeres on the third day after insemination, average diameter of blastocyst, antral follicle count, existence of immune sterility, existence of oviduct infertility, existence of endometriosis, insemination procedures, ovarian stimulation method, grade of smooth endoplasmic reticulum cluster, degree of blastocyst expansion, presence of vacuoles, refractile body, male age, and male body mass index, were collected to evaluate the outcome of implantation versus non‐implantation (Table 2). This information was acquired by physicians and embryologists who have been engaged in clinical practice for over 20 years and implemented standard laboratory practices related to embryo morphological evaluations in accordance with the relevant 2011 international consensus meeting.^16^

**TABLE 2:** Comparison of implantation and non‐implantation for nine conventional embryo evaluation factors and morphological features and clinical information of 977 blastocysts and univariate regression formulas of the independent factors for predicting implantation probability.

Item	Outcome	Mean ± SD	Min	Max	Median	*p*‐value of Mann–Whitney test	Formulas	Coefficients
Female age	Implantation	34.0 ± 4.4	20	46	34			
	Non‐implantation	36.6 ± 4.67	22	46	37	3.71 × 10^−18^		
	Total	35.36 ± 4.73	20	46	35		*k*/(1 + Exp (*β*_0_ + *β*_1_ *x*))	*β*_0_ = −12.112 ± 8.992, *β*_1_ = 0.286 ± 0.217, *k* = 0.506
Number of previous embryo transfer procedures	Implantation	2.16 ± 2.05	1	23	1			
	Non‐implantation	2.94 ± 2.42	1	13	2	8.22 × 10^−11^		
	Total	2.57 ± 2.29	1	23	2		1/(1 + Exp (*β*_0_ + *β*_1_ *x*))	*β*_0_ = −0.2712 ± 1.245, *β*_1_ = 0.166 ± 0.175
Anti‐Müllerian hormone concentration (ng/mL)	Implantation	3.29 ± 2.84	0.01	22.5	2.575			
	Non‐implantation	2.75 ± 2.81	0.05	22.96	1.93	2.51 × 10^−6^		
	Total	3.00 ± 2.83	0.01	22.96	2.26		1/(1 + Exp (*β*_0_ + *β*_1_ *x*))	*β*_0_ = 0.358 ± 2.068, *β*_1_ = −0.046 ± 0.158
Day 3 blastomere numbers	Implantation	8.72 ± 2.03	4	14	8			
	Non‐implantation	8.42 ± 1.99	3	16	8	0.025		
	Total	8.56 ± 2.01	3	16	8		*k*/(2*πσ* ^2^)^1/2^ Exp (−(*x*‐m)^2^/(2*σ* ^2^))	*σ* = 4.813 ± 1.142, m = 10.063 ± 0.874, *k* = 6.737 ± 1.259
Grade on day 3 (class A: 1; B: 2; C: 3; D: 4)	Implantation	1.93 ± 0.44	1	3	2			
	Non‐implantation	2.00 ± 0.49	1	3	2	0.03		
	Total	1.97 ± 0.47	1	3	2		1/(1 + Exp (*β*_0_ + *β*_1_ *x*))	*β*_0_ = −8.381 ± 16.265, *β*_1_ = 2.574 ± 5.645, *k* = 0.500
Embryo cryopreservation day (day 5: 1; day 6: 2; day 7: 3)	Implantation	1.31 ± 0.47	1	3	1			
	Non‐implantation	1.5 ± 0.54	1	3	1	3.07 × 10^−8^		
	Total	1.41 ± 0.51	1	3	1		*β*_0_ + *β*_1_ *x*	*β*_0_ = 0.852, *β*_1_ = −0.161
Inner cell mass (A: 1; B:2; C: 3)	Implantation	1.45 ± 0.53	1	3	1			
	Non‐implantation	1.72 ± 0.58	1	3	2	2.11 × 10^−13^		
	Total	1.59 ± 0.57	1	3	2		*β*_0_ + *β*_1_ *x*	*β*_0_ = 0.789 ± 0.011, *β*_1_ = −0.200 ± 0.005
Trophectoderm (A: 1; B:2; C: 3)	Implantation	1.53 ± 0.69	1	3	1			
	Non‐implantation	1.98 ± 0.79	1	3	2	2.92 × 10^−19^		
	Total	1.77 ± 0.78	1	3	2		*β*_0_ + *β*_1_ *x*	*β*_0_ = 0.791 ± 0.018, *β*_1_ = −0.182 ± 0.008
Average diameter of blastocyst (μm)	Implantation	187.8 ± 25.45	125.1	371.5	188.1			
	Non‐implantation	174.6 ± 27.16	119.5	316.9	174	1.32 × 10^−15^		
	Total	180.8 ± 27.17	119.56	371.59	181.17		1/(1 + Exp (*β*_0_ + *β*_1_ *x*))	*β*_0_ = 0.308 ± 4.242, *β*_1_ = −0.001 ± 0.016

*Note*: Each formula was determined to fit the data distribution. Coefficients are shown as the mean ± standard error.

Abbreviation: SD, standard deviation.

Because we thought bias of raw data could be reduced by using a regression function,^9^ we used univariate regression functions with maternal age, past embryo transfer time, anti‐Müllerian hormone value, blastocyst grade on day 3, embryo cryopreservation day, trophoblast grade, inner cell mass grade, number of blastomeres on the third day after insemination, and average diameter of blastocysts, respectively, selected as independent factors without multicollinearity, which indicated a state of strong correlations.

### 2.3 Blastocyst images

After an intracytoplasmic sperm injection procedure, or the day after insemination and oocyte denudation, the oocytes were immediately placed in culture slides containing 25 micro‐wells with a 280‐μm diameter (LinKID™ micro25, Dai‐Nihon‐Shoji Co., Ltd., Tokyo, Japan), covered with mineral oil, and incubated in a time‐lapse incubator system (CCM‐iBIS‐SL™, Astec Co., Ltd., Fukuoka, Japan) at 37°C, 6.5% CO_2_, and 5% O_2_. Images were acquired at 15‐min intervals. The time‐lapse system automatically focused on the vertical center position of the blastocyst and took a total of 11 consecutive tomographic blastocyst images, upper and lower, at 10‐μm pitch intervals on day 5 mostly or on day 6 or 7 in some cases of which blastocyst seemed not to be large enough. Each image was captured using a 1.3‐million‐pixel charge‐coupled device camera and saved at a size of 364 × 364 pixels. The images were deidentified.

### 2.4 AI for eliminating background

The deidentified dataset included all CEE factors, images of the blastocysts that resulted in implantation, or no‐implantation were transferred to the AI system offline at Medical Data Labo (Okayama, Japan). The first AI system was created by using semantic segmentation to eliminate the image background.^17^ The neural net, ForkNet,^14^ was obtained from Wolfram Neural Net Repository.^18^ It was initialized then trained with 1172 datasets of blastocyst images to create the first AI system consisting of 12 convolutional layers with multiple kernel sizes,^19^, ^20^, ^21^ 17 batch normalization layers,^22^ 5 catenate layers,^23^ 10 element‐wise layers,^24^ six pooling layers,^25^, ^26^, ^27^, ^28^ one logistic sigmoid layer.^29^ The first AI system was evaluated with 146 dataset of blastocyst images that were not overlapped with the training datasets. Then, this first AI was applied to all blastocyst images so that the background of each blastocyst image was eliminated and the mean intersection over union (mIOU) that is an evaluation metric used to measure the accuracy of an object detector on a particular dataset^30^ for eliminating the background of each blastocyst image was calculated, and then was saved at 111 × 111 pixels because of the hardware restrictions in this study.

### 2.5 Preparation for AI classifier

The consecutive tomographic blastocyst images for which the background was eliminated by the first AI system were transformed to matrix data of real numbers, then combined as a 3D model.

### 2.6 AI classifier for evaluating implantation

**FIGURE 1:**
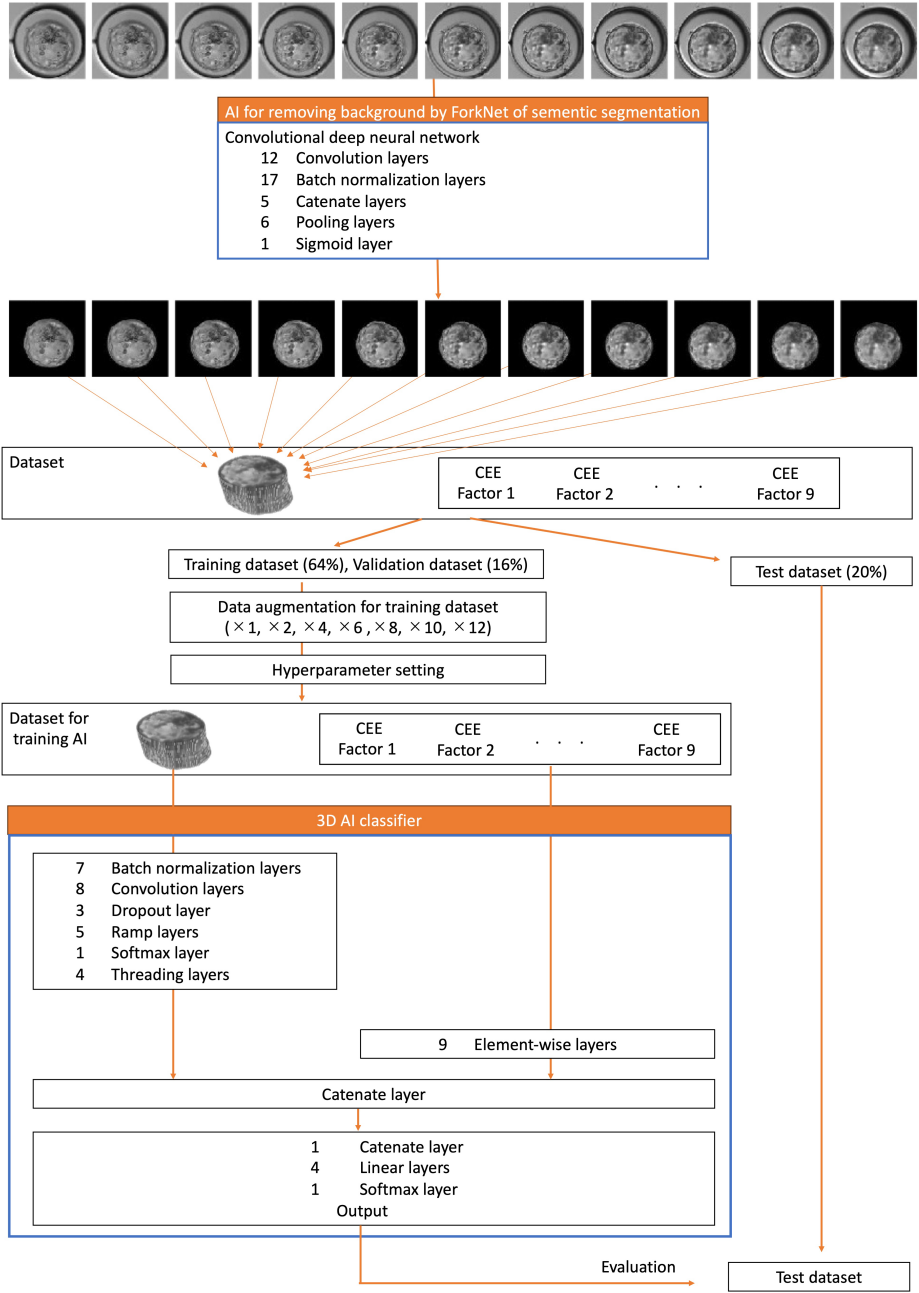
Flowchart for generating the artificial intelligence (AI) classifiers. The first AI system was created by transfer learning using semantic segmentation to eliminate the image background. The consecutive tomographic blastocyst images for which the background was eliminated by the first AI system were transformed to matrix data of real numbers, then combined as a 3D model. The nine CEE factors chosen as independent factors to predict implantation were age, number of embryo transfers, anti‐Müllerian hormone concentration, day 3 blastomere number, grade on day 3, embryo cryopreservation day, inner cell mass, trophectoderm, and average diameter. The 3D‐AI classifier was an AI classifier for a 3D model. The functions in the elementwise layer for each CEE factor are shown as formulas in Table 2.

Original AI classification programs were developed as shown in Figure 1. AI classifiers comprising both a convolutional neural network (CNN)^31^, ^32^, ^33^, ^34^, ^35^, ^36^ with batch size of 64, learning rate of 8 × 10^4^ and L2 regularization as a hyperparameter^37^, ^38^ and elementwise functions that apply a function to each element of a tensor for each CEE factor to obtain the probability of predicting an implantation or non‐implantation (Table 2). We introduced deep learning for the 3D models, with an original CNN architecture. The CNN comprised 36 layers: eight convolutional layers, seven batch normalization layers, two catenate layers, nine element‐wise layers, four linear layers,^39^, ^40^ five rectified linear layers,^41^, ^42^ and one threading layer.^43^ We also created elementwise functions for this study's CEE factors by regression analysis.

Then, a vector from 3D image input to the CNN, and scalers by the elementwise functions for the factors of the CEE were catenated. Finally, the data were placed in a softmax layer,^44^, ^45^ which presented the confidence score of the implantation.

We investigated the appropriate number of training datasets by evaluating the AUC value using the fivefold cross‐validation method.^46^, ^47^, ^48^ First, one‐fifth of all data were used as the AI test dataset. Then, the remaining four‐fifths of the data were divided into five datasets, with one‐fifth of the dataset being the validation dataset and four‐fifths of the dataset being the training dataset, respectively. The AI training dataset, validation dataset, and non‐overlapping test dataset were created in this fashion. The AI classifier was trained by an AI training dataset with concurrent validation by the validation dataset. Then, the AI classifier was evaluated with the test dataset. The training dataset was augmented by rotating images, as is often performed in the AI classifier process known as data augmentation. This is because blastocyst image processing with any degree of rotation can produce images resulting in different vector data for the same category.^9^, ^10^ The influence of L2 regularization and the appropriate data augmentation of the training dataset were investigated, and then sufficient 3D‐AI were generated under appropriate conditions for L2 and data augmentation with the early stopping rule. Finally, the AI classifier showing the best AUC value was obtained. Using the identical test dataset, an AI was created from only the sixth central image from the 11 tomographic images for comparison with the 3D‐AI, and the diagnostic performance of the 3D‐AI on reconstructed 3D image data from 11 images without eliminating background was also examined. The AUC values across Society for Assisted Reproductive Technology (SART) age groups were compared with the test dataset.

### 2.7 Development environment

The tools and conditions for development used were AMD Ryzen 9 5900X with 64 GB (Santa Clara, CA, USA) running Microsoft Windows 11 (Redmond, WA, USA), with NVIDIA GeForce RTX 3090 (Santa Clara, CA, USA) and Wolfram Language 13.1 (Wolfram Research, Champaign, IL, USA).

### 2.8 Statistical analysis

Wolfram Language 13.1 was used for all statistical analyses, using DeLong statistical test,^49^ Mann–Whitney *U*‐test, logistic regression analysis and univariate regression analysis. *p* < 0.05 indicated statistical significance.

## 3 RESULTS

Implantation and non‐implantation cases numbered 458 and 519, respectively. A total of 10 747 images were obtained. The CEE values were as follows; 35.36 ± 4.73 as female age, 2.57 ± 2.29 as number of previous embryo transfers procedures, 3.00 ± 2.83 (mean ± standard deviation [SD]) as anti‐Müllerian hormone concentration (ng/ml), 8.56 ± 2.01 as day 3 blastomere numbers, 1.97 ± 0.47 as grade on day 3 (class A: 1; class B: 2; class C: 3; class D: 4), 1.41 ± 0.51 as embryo cryopreservation day (day 5: 1; day 6: 2; day 7: 3), 1.59 ± 0.57 as inner cell mass (A: 1; B: 2; C: 3), 1.77 ± 0.78 as trophectoderm (A: 1; B: 2; C: 3) and 180.8 ± 27.17 as average diameter (μm). The number of embryo cryopreservation was 586, 380, and 11 for days 5, 6, and 7, respectively. Each factor was independently and significantly associated with implantation (Table 2). As for age distribution, the number of implantation/total was 252/423 for <35 (year old), 99/199 for 35–37, 77/194 for 38–40, 10/55 for 41–42, 20/106 for ≥42, respectively.

### 3.1 Univariate regression functions

The univariate regression functions for using at the elementwise layer in the neural network were as follows: Age, *k*/[1 + Exp (*β*_0_ + *β*_1_ *x*)], *β*_0_ = −12.112 ± 8.992, *β*_1_ = 0.286 ± 0.217, *k* = 0.506; number of embryo transfers, 1/[1 + Exp (*β*_0_ + *β*_1_ *x*)], *β*_0_ = −0.2712 ± 1.245, *β*_1_ = 0.166 ± 0.175; anti‐Müllerian hormone concentration (ng/mL), 1/[1 + Exp (*β*_0_ + *β*_1_ *x*)], *β*_0_ = 0.358 ± 2.068, *β*_1_ = −0.046 ± 0.158; day 3 blastomere number, *k*/(2*πσ* ^2^)^0.5^ Exp [(*x* − *m*)^2^/(2*σ* ^2^)], *σ* = 4.813 ± 1.142, *m* = 10.063 ± 0.874, *k* = 6.737 ± 1.259; grade on day 3 (class A: 1; B: 2; C: 3; D: 4), *k*/[1 + Exp (*β*_0_ + *β*_1_ *x*)]; *β*_0_ = −8.381 ± 16.265, *β*_1_ = 2.574 ± 5.645, *k* = 0.500; embryo cryopreservation day (day 5 = 1, day 6 = 2), *β*_0_ + *β*_1_ *x*; *β*_0_ = 0.852, *β*_1_ = −0.161; inner cell mass (A: 1; B: 2; C: 3), *β*_0_ + *β*_1_ *x*, *β*_0_ = 0.789 ± 0.011, *β*_1_ = −0.200 ± 0.005; trophectoderm (A: 1; B: 2; C: 3), *β*_0_ + *β*_1_ *x*; *β*_0_ = 0.791 ± 0.018, *β*_1_ = −0.182 ± 0.008; and average diameter (μm), 1/[1 + Exp (*β*_0_ + *β*_1_ *x*)], *β*_0_ = 0.308 ± 4.242, *β*_1_ = −0.001 ± 0.016 (Table 2).

**FIGURE 2:**
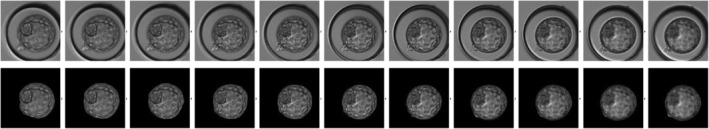
Example of a sequence of images of a blastocyst on day 5 taken with a time‐lapse device (upper panel). The blastocyst is in a 280‐μm diameter well and is surrounded by a frame. The lower panel shows a sequential image of a blastocyst with the background eliminated.

Figure 2 shows examples of sequential images of blastocysts taken with a time‐lapse device and their backgrounds eliminated by the first AI system.

### 3.2 The AI for removing background

The mIOU for eliminating the background of blastocyst images was 0.939 ± 0.033 (mean ± SD). The IOU value of the minimum and the maximum were 0.781 and 0.987, respectively.

### 3.3 The 3D‐AI classifier

**FIGURE 3:**
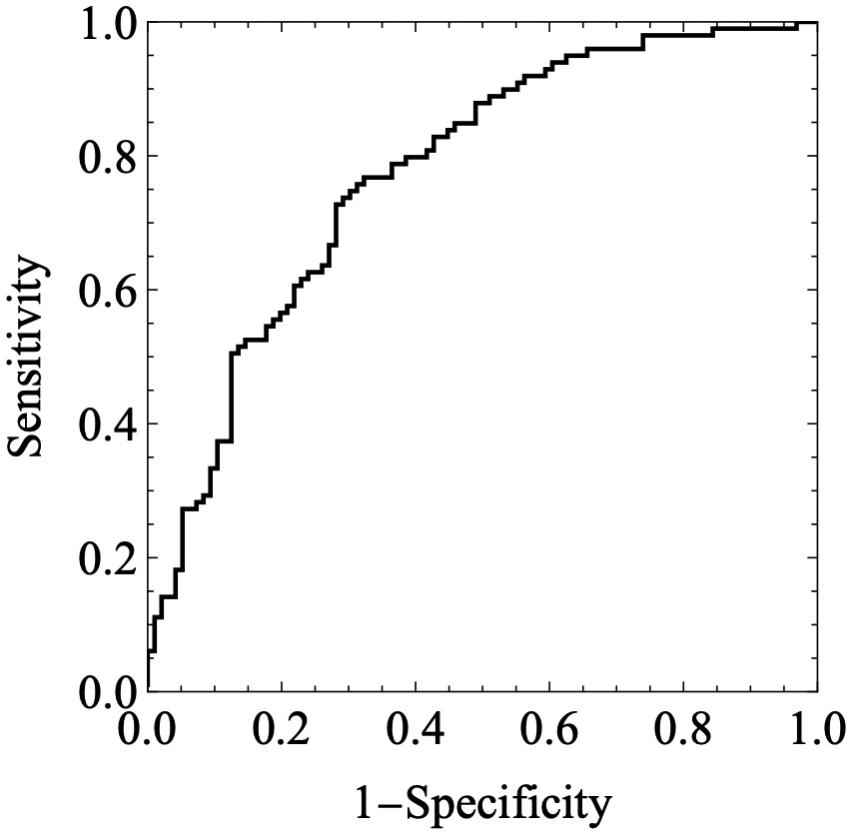
The receiver operator characteristic curve for implantation by the dual artificial intelligence system. The area under the curve was 0.774 ± 0.033 (mean ± standard error). The cut‐off point was 0.471.

**FIGURE 4:**
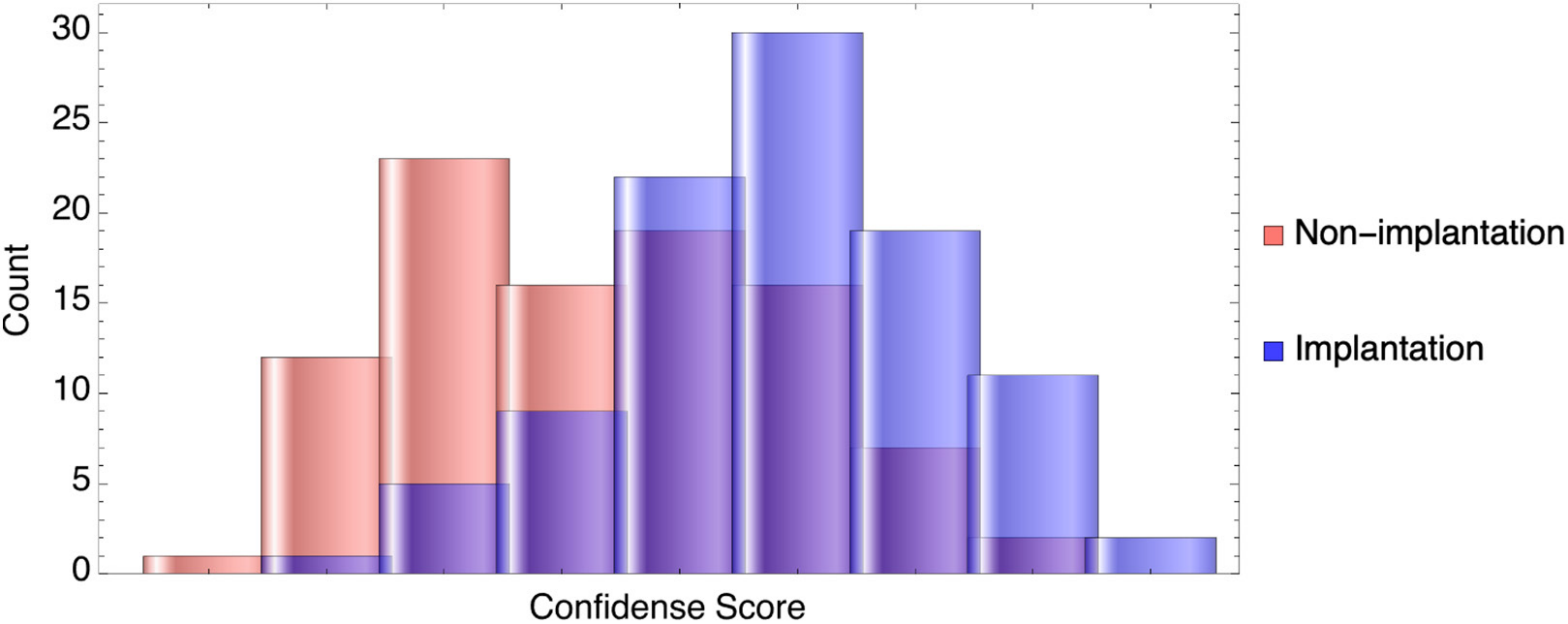
The distribution of the model's confidence scores separately for implantation and non‐implantation cases. The score was 0.539 ± 0.139 (Mean ± Standard deviation) and 0.381 ± 0.158 for implantation and non‐implantation cases, respectively. There is significant difference of the two groups (*p* = 3.77 × 10^−11^ by Mann–Whitney *U*‐test).

The best performance of the 3D‐AI classifier was obtained under an L2‐regularization value of 0.02 and the data augmentation was 10 times of the training data shown in Tables 3 and 4 and Figure 3. Data augmentation significantly affected AUC values in the two‐way analysis of variance (ANOVA) test, *p* = 2.166 × 10^−7^, but L2‐regularization value did not (*p* = 0.130). The dual AI system demonstrated 0.774 ± 0.033, 0.709–0.840 (mean ± standard [SE], 95% confidence interval) for AUC, 0.727 for sensitivity, 0.719 for specificity, 0.727 for predictive value of positive test, 0.719 for predictive value of negative test and 0.723 for accuracy with 0.471 as a cut‐off point. The result of the AI is shown in Table 4. As shown in Figure 4, the confidence score was 0.539 ± 0.139 (Mean ± SD) and 0.381 ± 0.158 for implantation and non‐implantation cases, respectively. There is significant difference of the two groups (*p* = 3.77 × 10^−11^ by Mann–Whitney *U*‐test).

**TABLE 3:** Highest‐performing three‐dimensional artificial intelligence results for determining pregnancy success from blastocysts.

Statistic	Value
AUC^a^ ± standard error	0.774 ± 0.033
95% confidence interval of AUC	0.709–0.840
Sensitivity	0.727
Specificity	0.719
Predictive value of positive test	0.727
Predictive value of negative test	0.719
Accuracy	0.723

^a^ Area under the characteristic curve.

**TABLE 4:** Result of classifying test dataset by 3D‐AI.

	Implantation by AI	No implantation by AI	Total
Implantation	72	27	99
No implantation	27	69	96
Total	99	96	197

As shown in Table 5, the AUC value and 95% CI of AI with almost the same architecture as the 3D‐AI, as for only one tomographic image instead of 11 images was 0.718 ± 0.036 (mean ± SE) and 0.646–0.789. The reconstructed 3D image as input data was better than an image but not significant by DeLong statistical test (*p* = 0.059). The AUC value and 95% CI of the 3D‐AI for 11 images without the removal of the background was 0.691 ± 0.038 (mean ± SE) and 0.618–0.765. The removal of the background was significant by DeLong statistical test (*p* = 0.011).

**TABLE 5:** Comparison of the AUC results of background elimination (with and without) and the comparison between using 11 images versus a single image in this study.

Method	AUC for predicting implantation (mean ± SE)	DeLong statistical test
AI for eliminating background and AI for 3D reconstructed image	0.774 ± 0.033	N/A
AI for eliminating background and AI for a single image	0.718 ± 0.036	*p* = 0.059
AI for 3D reconstructed image without background elimination	0.691 ± 0.038	*p* = 0.011

*Note*: The reconstructed 3D image was better than single images, though it was marginally insignificant (*p* = 0.059 by DeLong statistical test). The AI with the background eliminated showed a higher AUC value (*p* = 0.011).

Abbreviations: N/A, not available; SE, standard error.

The AUC values by the 3D‐AI across SART age groups were 0.722 ± 0.052 (mean ± SE) for <35 (year old), 0.742 ± 0.078 for 35–37, 0.716 ± 0.086 for 38–40, 1.000 ± 0.000 for 41–42, 0.647 ± 0.312 for ≥42, respectively. There was no differences among age groups (*p* = 0.492).

## 4 DISCUSSION

We created a high‐performance system that predicts implantation using blastocyst 3D images and CEE using dual AI. After eliminating the background of blastocyst tomograms by the semantic segmentation method with the mIOU value as 0.939 that seemed to be good, the dual AI system demonstrated 0.774 ± 0.033, 0.709–0.840 (mean ± SE) for AUC. Moreover, there was no difference among age groups regarding the AUC values. Because the results from published reports described that the AUC values demonstrated by various kinds of single AI system were 0.70,^7^ 0.71,^3^ 0.736,^2^ 0.74,^5^ 0.75,^6^ and 0.662–0.759 classified by age,^4^ the dual AI system demonstrating the good AUC might be useful. The sensitivity, specificity, predictive value of positive test, predictive value of negative test and accuracy were 0.727, 0.719, 0.727, 0.719, and 0.723, respectively (Table 3). The balance between cases where the implantation was successful and cases where it was not likely affected the positive and negative predictive values, which seemed to show the reliability of interpreting the results.

The good results might owe to the combination of background elimination, 3D data, and multimodality that was simultaneous learning of image information and CEE. Regarding background elimination, it is generally accepted that if a large amount of data is used for training by deep learning, it is unnecessary to eliminate the background as noise, such as the frame of the container. However, our experiment compared the performance of AI using blastocyst tomograms with and without the background, and found the AI with the background eliminated showed a higher AUC value (*p* = 0.011). Availability of more data may render it unnecessary to eliminate the background, but eliminating information irrelevant to the diagnosis, if possible, would still be desirable. Additionally, background elimination would be desirable for 3D reconstruction as in this study, considering that the background is also 3D.

As an input format, reconstructed 3D data were better than single images, though it was marginally insignificant (*p* = 0.059). Regarding 3D data, stereoscopic image data, in which vertical connectivity is reliably maintained, can naturally be considered a better subject for analysis than planar image data. Although classical microscopy could not image tomography in the depth direction with accurate slice widths, the development of time‐lapse instruments enables reconstruction of 3D data.

Regarding multimodality, we have reported the feasibility of AI combining a blastocyst image and CEE for predicting live birth, resulting in the AUC value of 0.740 ± 0.031 (mean ± SE) with using a total of 5691 blastocyst planar images (2019).^12^ We have also reported that the prediction for live birth calculated by the multivariate logistic function combining the AI prediction from a planar image and the prediction from CEE performed better than the value predicted by the AI from the planar image alone or by the multivariate logistic function of CEE alone (2019).^10^ Therefore, a diagnostic AI system incorporating images and CEE seemed to be desirable. The dual AI system in this study, which uses background‐removed 3D reconstructed blastocysts incorporating CEE, could be considered an extension and an advanced version of our previous AI system that used a planar image incorporating CEE.

The data augmentation that significantly affected the AUC values was considered a necessary procedure. But L2‐regularization for avoiding over‐fitting for the 3D‐AI was not a significant hyperparametric factor in this study. This might be due to the function of the batch normalization layer, dropout layer, etc., that were included in the AI architecture itself for avoiding over‐fitting.

There were some limitations in this study. First, a time‐lapse device with a slice imaging function is necessary to obtain tomographic images with accurate slice information. If such a device is not available, tomographic images can be collected only by manual imaging, which is less accurate. Additionally, the AUC value of 0.774 obtained from the three‐dimensional reconstructed images created from 11 consecutive tomographic blastocyst images suggested useful, but it is unclear whether the image data size was sufficient. Therefore, more slices or a narrower slice pitch might improve the performance, as should further applying the whole size of the blastocyst. Second, if new parameters that are unknown such as biomarker information would be discovered, the CEE might need to be changed. The 3D‐AI will need to be re‐created if that occurs. Third, when the dual AI system would be used in other facilities, this study was conducted with data from a single institution only resulting in possible domain shift problem that is a major problem that might negatively affect the performance in other domains.^50^ Verification is needed before it can be applied to other facilities. Fourth, there may be the possibility of device specific domain shift arising from the use of a single type of device. The use of data from only one type of time‐lapse incubator may affect the generalizability of the AI system to data from different devices or manufacturers. Verification is also needed before it can be applied to other devices or manufacturers. Finally, it is unclear whether this study used enough the number of patients to create the dual AI system, but as data augmentation affected the AUC, a further increase in data might improve AUC values.

From a practical standpoint, it is currently possible for a medical institution to upload multiple blastocyst images and CEE data to an Internet‐based cloud server, and the dual AI system will analyze the predicted probability of implantation of each blastocyst by the latest AI, even if improved in the future. The results can be automatically sent back to the institution via email and the predicted implantation probability values for each blastocyst could be used as a reference to help doctors and embryologists in selecting a blastocyst order for transfer.

In the present case, 3D reconstruction was performed from the blastocyst at the time when it was determined as just before freezing. However, as time‐lapse observation is possible from the early stages of culture, time‐series data of 3D reconstruction (i.e., 4D data) can be obtained. Direct or indirect analysis of such 4D data can be called 4D‐AI. In the future, research on 4D‐AI with CEE or AI that handles dimensional data with more than four dimensions can be expected to develop. As methods have been reported to compare past, present, and future blastocyst classifying methods, if proper data storage is available,^51^ appropriate selection of various AI systems, including 3D‐AI, will be possible in the future.

Our dual AI system using blastocyst 3D images from the time‐lapse device and CEE will enable probabilistic prediction of implantation and will indicate the order of embryo transfer selection. This should be useful from the standpoints of time, economy, and mental health for patients who wish to conceive.

## CONFLICT OF INTEREST STATEMENT

Yasunari Miyagi, Toshihiro Habara, Rei Hirata, and Nobuyoshi Hayashi declare that they have no conflicts of interest.

## ETHICAL APPROVAL

The protocol for this research project including the use of patients was approved by the IRB at Okayama Couples' Clinic (IRB no. 2022–09).

## HUMAN RIGHTS STATEMENTS AND INFORMED CONSENT

All procedures followed were performed in accordance with the ethical standards of the responsible committee on human experimentation and with the Helsinki Declaration of 1964 and its later amendments. Informed consent was obtained from all patients for inclusion. We obtained additional informed consent from all patients for whom identifying information is included. A Web site with additional information including an “opt‐out” option was set up for this study.

## ANIMAL STUDIES

No animals were used in this study.

## CLINICAL TRIAL REGISTRY

This study is not a clinical trial.
